# Barriers and Strategies to Lifestyle and Dietary Pattern Interventions for Prevention and Management of TYPE-2 Diabetes in Africa, Systematic Review

**DOI:** 10.1155/2020/7948712

**Published:** 2020-07-11

**Authors:** Hirut Bekele, Adisu Asefa, Bekalu Getachew, Abebe Muche Belete

**Affiliations:** ^1^Department of Nursing, Institute of Health Sciences, Bethel University, USA; ^2^Department of Pre-clinical, Institute of Medicine and Health Sciences, Debreberhan University, Ethiopia; ^3^Department of Biomedical Science, Institute of Health Sciences, Jimma University, Ethiopia

## Abstract

**Background:**

Diabetes mellitus is a major chronic illness in Africa that requires lifelong lifestyle interventions and pharmacological therapy. Lifestyle change is the most important aspect of diabetes care and includes diabetes self-management education and support, medical nutrition therapy, physical activity, smoking cessation counseling, and psychosocial care.

**Purpose:**

The purpose of this project was to review published articles that investigate lifestyle and dietary pattern interventions for diabetes prevention and management in Africa. Barriers to lifestyle interventions and strategies to overcome the barriers are also reviewed in this study.

**Methods:**

The article search was conducted in an electronic database search of PubMed, Google Scholar, and Cochrane Library. Studies were included if they were published between 2011 and 2019, if they were conducted in an African country, and were written in the English language.

**Results:**

Articles reviewed included several that examined the basic lifestyle and dietary pattern changes for all patients diagnosed with type 2 diabetes, on self-care behavior of type 2 diabetes patients, on the cost of diabetes in Africa, and on barriers for adherence to lifestyle and dietary changes in Africa, with strategies to address those barriers.

**Conclusion:**

Lifestyle interventions including regular physical exercise, weight management, and adherence to health care professionals' recommendations on a healthy diet are the cornerstone in the prevention and management of diabetes in Africa. The main barriers to adherence were both systemic (population changes, poor access, western cultural influences, and low-quality healthcare) and personal (poverty and cost, educational status, and perceptions about the disease) in nature. The strategies for the barriers include health education programs, advocacy, and capacity building.

## 1. Introduction

Diabetes mellitus is an endocrine disorder with heterogeneous etiologies, which is characterized by raised levels of glucose in a person's blood and disturbances of macromolecules such as carbohydrate, fat, and protein metabolism resulting from defects in insulin secretion, insulin action, or both. A lack of insulin, or the inability of cells to respond to it, leads to high levels of blood glucose (hyperglycemia), which is the clinical indicator of diabetes [1]. Type 1 DM is characterized by insulin deficiency and a tendency to develop diabetic ketoacidosis, whereas type 2 DM is characterized by variable degrees of insulin resistance, impaired insulin secretion, and excessive hepatic glucose production [2].

Diabetes is one of the most rapidly increasing chronic diseases and an important public health problem all over the world. The global burden of diabetes is rising dramatically worldwide. According to the 2019 International Diabetes Federation (IDF) report, approximately 463 million adults (20-79 years) were living with diabetes. This represents 9.3% of the world's population in this age group. The total number is predicted to rise to 578 million (10.2%) by 2030 and to 700 million (10.9%) by 2045. The number of deaths resulting from diabetes and its complications in 2019 is estimated to be 4.2 million. The proportion of people with type 2 diabetes is increasing in most countries. Among adult diabetes, 79% were living in low- and middle-income countries. Among age above 65 years, 1 in 5 has diabetes. It caused at least 760 billion dollars in health expenditure in 2019-10% of total spending on adults [3, 4].

Diabetes estimates for 2019 show a typically increasing prevalence of diabetes by age. Similar trends are predicted for the years 2030 and 2045. Prevalence is lowest among adults aged 20–24 years (1.4% in 2019). Among adults aged 75–79 years, diabetes prevalence is estimated to be 19.9% in 2019 and predicted to rise to 20.4% and 20.5% in 2030, and 2045, respectively. The estimated prevalence of diabetes in women aged 20–79 years is slightly lower than in men (9.0% vs. 9.6%). In 2019, there are about 17.2 million more men than women living with diabetes [3, 4].

Type 2 diabetes is the most common type of diabetes, accounting for around 90% of all diabetes worldwide (IDF). The prevalence of type 2 diabetes is high and rising across all regions. This rise is driven by increasing life expectancy, economic development, and increasing urbanization leading to more sedentary lifestyles and greater consumption of unhealthy foods linked with obesity [5].

Diabetes mellitus is currently a major challenging public health problem in Africa [6]. According to the 2019 IDF Africa report, approximately 19 million adults (20-79 years) are living with diabetes; representing a regional prevalence of 3.9%; by 2045 estimated to increase to 47 million. It caused at least 9.5 billion dollars in health expenditure in <1% of the total global expenditure on diabetes. More than half of these patients live in the following most populous African countries. Diabetes national prevalence (%) in adults 20–79 years in these African countries showed South Africa (12.8%), the Democratic Republic of Congo (4.8%), Nigeria (3%), and Ethiopia (3.2%) [3, 4]. The main factors that contribute to the rise of diabetes in Africa are epidemiological transition, population, and lifestyle changes [7]. In 2019, 366,200 deaths (6.8% of all-cause mortality) in the AFR Region are attributable to diabetes with the highest percentage (9.1%) of all-cause mortality due to diabetes in the age group 30–39 years. People below the age of 60 accounted for 73.1% of these deaths in Africa [3, 4].

Diabetes is the fourth leading cause of mortality in the world and accounts for 80% of the deaths that occur in developing countries. According to a report by the WHO, diabetes-related deaths will continue to rise significantly in Africa if no immediate action is taken. Type 2 diabetes mellitus (T2DM) has also resulted in a massive economic challenge for the developing countries, including those in Africa [8]. The cost of diabetes care and treatment is expensive, which creates an economic burden to the already weak economy of these countries. In developing countries where there are scarce resources to address diabetes mellitus and to create awareness in diabetes patients, it is important to take immediate action for the implementation of new programs like an intensive lifestyle intervention like goal-based behavioral intervention (weight loss goal, self-monitoring of weight, dietary modification, self-monitoring fat and/or calorie intake, and physical activity goal) and case managers or “lifestyle coaches” to deliver the intervention to prevent and manage complications related to T2DM [9].

Patients with diabetes are prone to developing chronic illnesses such as cardiac disease, eye disease, renal disease, and diabetic foot complications [3, 4]. As the prevalence of diabetes increases, the complications associated with the disease will be very challenging to manage, consuming large amounts of resources and portion of healthcare budgets [10]. The long-term complications of diabetes can be present at diagnosis in people with type 2 diabetes. Type 2 diabetes is associated with cardiovascular conditions that collectively constitute the largest cause of both morbidity and death for people with diabetes (1). Diabetic eye disease (DED) is a much-feared complication of diabetes, consisting predominantly of diabetic retinopathy (DR), diabetic macular oedema (DMO), cataract, and glaucoma. In most countries, diabetic retinopathy is one of the major causes of blindness in the productive age group with devastating personal and socioeconomic consequences, despite being potentially preventable and treatable [11, 12]. Chronic kidney disease (CKD) in people with diabetes can result from diabetic nephropathy or can be the result of other associated conditions such as hypertension, polyneuropathic bladder dysfunction, increased incidence of relapsing urinary tract infections, or macrovascular angiopathy [13]. Globally, more than 80% of end-stage renal disease (ESRD) is caused by diabetes or hypertension, or a combination of both [14]. Diabetic foot and lower limb complications, which affect 40 to 60 million people with diabetes globally, are an important source of morbidity in people with diabetes. Chronic ulcers and amputations result in a significant reduction in the quality of life and increase the risk of early death [15].

The health care costs of managing the complications of diabetes account for over 50% of the direct health care costs of diabetes. Diabetes complications, as frequent causes of disability, premature mortality, and absence from work due to sickness, are important drivers of indirect costs. Type 2 diabetes and high body mass index are associated with an increased risk of a number of common cancers, with high body mass index associated with almost twice as many cancers as diabetes. The global rise in elevated body mass index and type 2 diabetes is cause for concern in relation to global cancer impact [3, 4]. The increase in morbidity and premature death due to diabetes create financial costs to individuals and to countries [16]. Management of diabetes is challenging for the overstretched healthcare systems in Africa [17]. The immediate need to address worldwide chronic diseases is currently one of the seventeen Sustainable Development Goals (SDG) [16]. The third goal of SDG explains a commitment to reduce premature death due to noncommunicable diseases (NCD) by one-third and to ensure universal health coverage by 2030 [16].

The cornerstone of type 2 diabetes management is the promotion of lifestyle interventions that include a healthy diet, regular physical activity, smoking cessation, and maintenance of a healthy body weight (9). “T2D is a lifestyle disease, which can and should be prevented by intensive lifestyle interventions, characterized by changes in dietary habits and increased physical activity” [18].

Changes in lifestyle and dietary patterns are essential to the management of T2DM. Lifestyle changes including regular physical exercise, weight management, and diet control help to mitigate the long-term effects of diabetes. Lifestyle intervention and education are important aspects of diabetes care for the health care team and include diabetes self-management education and support (DSMES), medical nutrition therapy (MNT), physical activity, smoking cessation counseling, and psychosocial care [19]. “DSMES is the ongoing process of facilitating knowledge, skill, ability and motivation for diabetes self-care that involves active participation of the people living with diabetes” [19]. “Medical nutritional therapy (MNT) is essential for diabetic prevention and management. The aims of MNT are to: - achieve individual glycemic, blood pressure and lipid goals, maintain body weight goals, and delay or prevent complications of diabetes” [19]. Therefore, type-2 diabetes-related complications and mortality are primarily prevented and controlled through adherence to healthy lifestyle habits. This can especially be a problem in developing countries where there is poor access and low quality of health care [20].

## 2. Methods

To find as many relevant articles as possible, a wide range of electronic databases were searched in order to identify articles on the influence of lifestyle and dietary pattern interventions in the prevention and management of type 2 diabetes in Africa. Articles that discussed barriers to self-care management using the lifestyle and dietary changes necessary were examined, as well as potential strategies for overcoming those barriers.

### 2.1. Description of Search Strategies

The article search was conducted in an electronic database search of PubMed and Cochrane Library. Additional searches were conducted via Google Scholar to investigate studies that may not be published in electronic databases. The main search terms for this review were the following: *Type-2 Diabetes Mellitus*, *Africa*, *Life Style Intervention*, *Diet, Diabetes Prevention and Management*, *Self-care Practices*, *Barriers*, *Strategies*.

After the studies were searched, the next step was removing redundant studies. In the first level of identification, the titles of the articles were examined to select studies to be included in this review. Reading the title of the article alone was not enough to determine whether the article was important with respect to the practice questions, so the article's abstracts were then examined. The final step involved was examining for eligibility through reading all the articles to determine which would be included and which would be excluded.

### 2.2. Criteria for Including or Excluding Research Studies

The inclusion and exclusion criteria for this review were determined via the PICOS (Population, Intervention, Comparison, Outcome, and Study design) guidance for conducting literature reviews [21]. Articles were included if they were published from 2011 to 2019 and were written in the English language. Many studies were available addressing diabetes disparities in the African immigrant population in the United States; however, this study focused mainly on diabetes in African countries. The rationale for the exclusion was that countries in Africa could provide the purest data affected the least by western cultural influences. Studies conducted on African populations that examined lifestyle interventions, dietary patterns, self-care practices, barriers for adherence, and strategies for the prevention and management of type-2 diabetes patients were included. Two systematic reviews conducted outside of Africa were also included since the findings from these articles were significant for the African population. Articles that did not fulfill the inclusion criteria were excluded.

### 2.3. Summary of Studies Selected for Review

**Figure 1:**
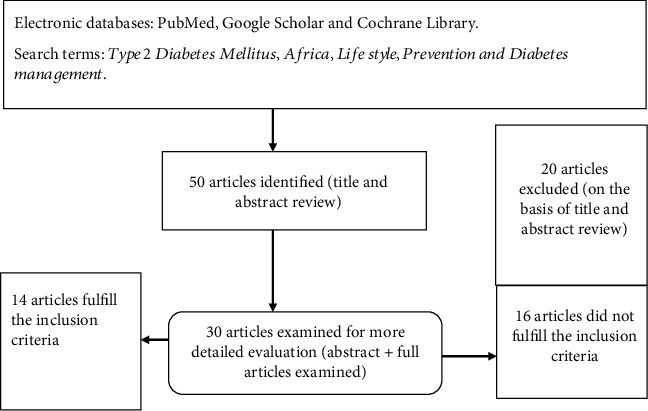
Diagram showing electronic databases, articles search, inclusion criteria, and selection process.

Fifty articles were identified via an electronic search of PubMed, Google Scholar, and Cochrane Library. After the titles and abstracts were reviewed, more detailed evaluations (abstract and full articles) were examined for thirty articles from these sixteen articles were excluded due to their older publication dates, and the fact that the studies were conducted in developed countries. As a result, fourteen articles were included in this review (see Figure 1).

### 2.4. Evidence Criteria for Research Studies

The levels of evidence and the quality guides of articles and research papers were evaluated based on the Johns Hopkins Method of Research Evidence Appraisal Tool [22]. According to this tool, the level of evidence ranges from level I to level V, and the quality guide ranges from high quality to low quality. Evidence level I includes articles conducted on randomized controlled trial (RCT), experimental study, and systematic review with or without meta-analysis. Evidence level II includes articles conducted on a systematic review of a combination of RCTs and quasi-experimental, quasi-experimental studies only with or without meta-analysis. Evidence level III includes studies conducted on nonexperimental study (cohort, crossectional, and other longitudinal studies), systematic review of a combination of nonexperimental studies, and RCTs quasi-experimental and/or nonexperimental studies only, with or without meta-analysis, and qualitative study. Evidence level IV includes the opinion of nationally recognized experts and/or respected authorities based on scientific background. Evidence level V, based on nonresearch evidence, includes literature reviews, case reports, quality improvement programs or financial evaluations, and experiential evidence (p. 1).

According to Johns Hopkins, “the quality guide ranges from high quality (consistent, generalizable results, sufficient sample size, adequate control group, definitive conclusions, and consistent recommendations) to low quality (little evidence, inconsistent results, insufficient sample size and difficulty of conclusion)” ([22], p.2).

Based on the Johns Hopkins Method of Research Evidence Appraisal Tool [22], the articles included in this review ranged from evidence level I to level III; according to the quality guide, they ranged from high to good quality.

## 3. Result and Analysis

### 3.1. The Matrix

The matrix (Table 1) is composed of fourteen articles that examine the prevalence of type 2 diabetes in Africa, barriers to lifestyle and dietary changes, and interventions for diabetes prevention and management. Articles on type 2 diabetes patient's self-care behavior were also included in the matrix. The Johns Hopkins Method of Research Evidence Appraisal Tool [22] was used to evaluate the levels of evidence and the quality guides of articles and research papers that were used.

### 3.2. Synthesis of Major Findings

Articles reviewed included several that discussed the definition and prevalence of type 2 diabetes in Africa, lifestyle and dietary pattern changes, and self-care behavior of type 2 diabetes patients. Articles on the cost of diabetes, barriers for adherence to lifestyle interventions, and strategies to address those barriers in Africa also reviewed. Four articles that outlined basic lifestyle and dietary pattern changes for all patients diagnosed with type 2 diabetes [23], (Rawal et al., 2013), [24, 25]; two articles on self-care behavior of type 2 diabetes patients [26, 27]; one article on the cost of diabetes in Africa [28], and seven articles that focused on barriers for adherence to lifestyle and dietary changes in Africa, and strategies to address those barriers [29–35]. The major findings of this critical review include dietary pattern changes, physical activity, and type 2 diabetes, and adherence to lifestyle modifications. The major barriers to poor adherence to lifestyle interventions and strategies are also examined in this section.

### 3.3. Lifestyle and Diet Changes and Interventions for Prevention and Management of Type 2 Diabetes in Africa

Lifestyle changes and interventions such as diabetes self-management education, social support, medical nutrition therapy, and physical activity are vital aspects of both diabetes prevention and diabetes management [19]. The significant findings of the articles showed that appropriate lifestyle interventions including regular physical exercise, an appropriate diet pattern, frequent blood glucose monitoring, and having support group improve the management of type 2 diabetes and reduce complications related to diabetes.

#### 3.3.1. Dietary Patterns

Dietary patterns have a high impact on the management of type 2 diabetes. Consideration of healthy diets such as vegetables, fruits, and a low-fat diet help to reduce the risk associated with diabetes. In general, plant food is associated with lower T2D risk than meat, low energy density food is considered more protective than high energy density food, associations of fish consumption with diabetes risk are variable, and fermented dairy products may be more beneficial than nonfermented ones [25]. The review results demonstrated that different dietary choices have been helpful in preventing and controlling diabetes. These different dietary choices include low-fat diets, low glycemic index diets, low carbohydrate diets, and Mediterranean diets. From these diets, the Mediterranean diet is the best for the health human being [36].

Ancel Keys introduced the Mediterranean Diet (MD) in the 1960s through observing the food habits of Mediterranean populations. “The Mediterranean diet is one of the best-known food patterns for human health. MD components consist of high intake of plant-based foods such as fruit, vegetables and legumes, moderate intake of fish and dairy products, and low intake of red meat and red wine” [37]. The Mediterranean diet has a significant outcome for the prevention and control of type 2 diabetes. It reduces oxidative stress, insulin resistance, serves as an anti-inflammatory dietary pattern, and decreases the complications of chronic diseases [38].

Nutritional therapy is an important component for diabetes prevention and management. Different studies showed that MNT reduces the HbA1c by 0.5 to 2%. “The objectives of MNT are to promote the enjoyment of a variety of nutrient-dense foods in appropriate portion sizes to: achieve individual glycemic, blood pressure and lipid goals, achieve and maintain body weight goals, delay or prevent complications of diabetes” [19].

The dietary patterns in Africa mainly consist of two diverse dietary patterns: a “purchase” dietary pattern that positively correlates with the consumption of sweets, rice, meat, fruits, and vegetables; and a “traditional” dietary pattern that correlates with the intake of fruits, plantains, green leafy vegetables, fish, fermented maize products, and palm oil. Traditional dietary patterns are inversely associated with type 2 diabetes. The influence of western culture resulted in a nutritional transition from traditional dietary patterns to high carbohydrate consumption and the emergence of prepackaged foods [33]. In addition, in most African countries, rice is a staple in the diet and served at every meal. Injera, which is a high carbohydrate food, is also the most dominant food that is served in Ethiopia and East African countries. So, even in a more traditional diet, changes need to be made in carbohydrate consumption in order to stay within diabetes prevention and management guidelines. The cultural diet and the eating habits of a society are difficult to change according to health professional diabetic diet recommendations [33].

#### 3.3.2. Physical Activity and Type 2 Diabetes

Physical exercise is another important intervention for the prevention and management of type 2 diabetes. “Regular physical activity helps the body cells take up glucose and thus lower blood glucose levels. Regular physical activity also helps with weight loss as well as controlling blood cholesterol and blood pressure” [36]. The beneficial effects of regular physical exercise in the management of diabetes include decreased weight gain, cholesterol levels, and blood triglycerides [36].

Several studies showed that practicing regular exercise has a significant effect on the survival of diabetes patients. “Prospective cohort and cross-sectional observational studies that assessed physical activity with questionnaires showed that higher physical activity levels are associated with reduced risk for type 2 diabetes, regardless of method of activity assessment, ranges of activity categories, and statistical methods” [39]. Several studies showed an association between physical inactivity and the incidence of type 2 diabetes [40]. Experimental studies showed that regular exercise together with a healthy diet helps to improve the blood glucose level [41].

Regular physical exercise has a beneficial effect to improve the physical, mental, and social health of diabetes patients. “Data showed that moderate exercise such as brisk walking reduces risk of type 2 diabetes, and all studies support the current recommendation of 2.5 h/week of a moderate aerobic activity or typically 30 min/day for 5 days/week for prevention” [39]. The intensity, duration, and frequency of recommended physical exercise is significant for reducing complication associated with diabetes.

In the past, walking was the main source of transportation in African culture. Currently, due to urbanization and Western influence, more people are using other modes of transport, thus decreasing the physical exercise that was a protective factor against diabetes.

### 3.4. Adherence to Lifestyle and Diet Pattern Modifications

According to the reviewed articles, adherence and nonadherence defined in relation to lifestyle and dietary patterns. Adherence is a collaborative relationship established between the patient and the healthcare provider, and the patient carefully follows the lifestyle modifications in which there is a mutual agreement that is beneficial for the patient. Nonadherence results when a diabetic patient does not follow the mutually agreed lifestyle and dietary changes [26].

The reviewed articles summarized multiple factors for nonadherence to lifestyle and dietary interventions; these includes the patient's educational status, income level, perceived severity, and perceived barriers [26, 29].

Several reviewed articles investigated the reason for the high rate of nonadherence to the recommended diet in type 2 diabetes patients. The proposed explanation for poor adherence to dietary recommendations was poor self-discipline, lack of awareness, eating outside home, lack of self-control, lack of recommended diet education, financial constraints to the recommended diet, eating an unhealthy diet at home when alone, poor attitude on the effectiveness of a diet to control blood glucose, lack of support from the partner, health professionals, family members, and friends, poor appetite for the recommended diet, and problems remembering the recommended diet were barriers to adherence to the recommended diet [26, 29, 32]. In addition, “Food habits in the family and personal food preferences were among the serious challenges that made dietary adjustment difficult for people with diabetes. Participation in social gatherings and food related socio-cultural norms could pose serious impediments to effective diabetic control in Sub-Saharan Africa. In addition shortage of cash to purchase food items appropriate for persons with diabetes, craving for cultural/traditional food and limited availability of variety of food items in the local market are barriers to dietary self-care practices” [27].

Several reviewed articles investigated the reason for the high rate of nonadherence to the recommended physical exercise in type 2 diabetes patients. The proposed explanation for poor adherence to physical exercise was poor knowledge, the perception that exercise potentially exacerbates illness, lack of an exercise partner, specific locations away from home, the rainy season in Africa, criticism by others, and lack of support from the partner, health professionals, family members, and friends [29, 32].

The result of reviewed articles showed the cost of managing diabetes mellitus as a potential explanation for nonadherence. The result of these reviewed articles indicated “the annual national direct economic costs of diabetes differed between countries. Indirect costs per patient for managing and controlling diabetes were generally higher than the direct costs per patient with diabetes. The most commonly listed healthcare costs were pharmaceutical costs, followed by diagnostic and investigation costs, medical equipment supply costs and consultation costs” [28].

The reviewed articles examined the cultural lens to understanding daily experiences with type 2 diabetes self-management among clinic patients. “Culture is a significant factor in shaping health behaviors such as diabetes self-management behavior, including exercise, medication, and dietary adherence. Chronic health conditions such as diabetes are shaped by cultural factors that influence perceptions of the disease, explanations of risk factors, and how people value and react to the disease and its symptoms and manifestations” [33].

### 3.5. Strengths and Weaknesses of the Research Studies

Extensive electronic database searches of PubMed, Google Scholar, and Cochrane Library were used. The tool for evaluating the articles for the included studies was the Johns Hopkins method of research evidence appraisal tool. The articles included in this review ranged from evidence level I to level III; according to the quality guide, they ranged from high to good quality. Most of the reviewed articles had reasonably consistent results, a sufficiently large sample size, for the RCT studies adequate control group, definitive conclusions, and consistent recommendations. RCTs were included in this review that is the gold standard for assessing an intervention. A study by Ayele et al. [30] explained the reason for poor adherence by using multivariate logistics regression analysis that is the best method of software analysis to examine the association between variables by excluding potential confounding factors. A study conducted by Rawal et al. [42] explained the keywords clearly. They used systematic review with RCTs which is evidence level one and high quality. They reviewed published articles. The included articles for the review were examined by two independent reviewers for eligibility. Any differences were resolved by discussion and consensus with other authors. The article evidence has shown reasonably consistent and positive results. A study by Mendenhall (2015) used a cohort study design. He recruited women from Birth to Twenty Plus (Bt20). He conducted in-depth interviews around issues of stress, diabetes, mental health, and diabetes care.

Several articles included in the review were high quality with a sufficiently large sample size. However, a few articles included in this review were small sample sizes that make it difficult to generalize the finding to a larger population. For example, a study done by [29, 34] included small sample sizes selected by using a convenience sampling method. This method poorly generalizes the finding of the study for large populations due to lack of random selection of patients.

In this review, only published articles were reviewed; this resulted in publication bias. The findings from unpublished articles may be different from published articles [43]. In this review, articles were included if they were published from 2011 to 2019. The findings from older articles might be different from recent articles. Few articles in this review were cross-sectional studies that were difficult to ascertain the association between the variables of exposure and outcome, since both variables were collected at the same time [29–32]. The findings from this design had little evidence, inconsistent results, insufficient sample size, and difficulty of conclusion. Finally, a study conducted by Ayele et al. [30] used a data collection tool that was adapted and modified from previous studies on similar topics. The data collection tool used in this study was not standardized.

## 4. Discussion

Diabetes mellitus is currently a major challenging public health problem in Africa. Even though the number of studies that describe the prevalence of diabetes in the Africa population was limited, there was a similarity in their findings showing an increased prevalence of diabetes in Africa.

### 4.1. Barriers to Lifestyle and Dietary Pattern Change and Intervention in Africa

The reviewed literature identified a number of barriers to lifestyle and dietary change adherence. Barriers were both systemic (population changes, poor access, Western cultural influences, and low-quality healthcare) and personal (poverty, cost, educational status, and perceptions about the disease) in nature. The most commonly explained barriers for poor adherence to lifestyle intervention in the order of importance included a lack of knowledge and education, poverty and cost, population changes, and lack of access to healthcare.

#### 4.1.1. Lack of Knowledge/Education

Lack of knowledge (poor awareness) regarding the effectiveness of modification of lifestyle change for diabetes prevention and management is common in most societies in Africa. “Lack of educational guidelines, shortage of human resource and time constraints are the main challenge to educate patients with chronic disease such as DM and HTN. It is also reported that difficulties of educating lifestyle interventions programs due to existing patients habits such as cultural diet and eating style in the region” [34].

A patient's educational status has been identified as an important barrier to optimal diabetes regimen adherence in the region. Patients with higher educational status have better adherence than those without education [29, 30]. Patients' knowledge and attitudes may also present a barrier. Some do not acknowledge the seriousness of the disease, and some do not correlate their diet and sedentary lifestyle as exacerbating their symptoms [29]. Misperception about the severity of the illness such as the complications associated with the disease contributes to poor adherence [32].

Patients' ideas, beliefs, and experiences with diabetes also greatly influence adherence. For example, there is a prevalent belief that exercise actually exacerbates the symptoms of diabetes, rather than being a protective factor [29]. From the patient's point of view, lifestyle modification recommendations in the management of type 2 diabetes are perceived to be time-consuming [29].

Finally, the knowledge, beliefs, and attitudes of family members and friends regarding dietary and lifestyle interventions positively correlate with adherence. Social support groups who have higher knowledge on the recommended lifestyle and dietary changes and interventions have a significant contribution to the management of the diseases [30]. Social support from partners, family members, and friends positively predicts adherence to diet and exercise recommendations [29]. “Family and friends play both supportive and obstructive roles. Participants identified both positive and negative contributions of family and community members to their diabetes management. Women were more likely to discuss struggles finding support compared to men” [33].

#### 4.1.2. Cost of Diabetic Care

The national costs of diabetes care in Africa were high, and it is unaffordable to the majority of the population. The diabetes-related direct costs differed between countries. “The difference in costs is due to differences in costing methods and cost categories included in the cost estimation, for example, the national direct costs of diabetes in Nigeria were estimated in the range of $3.5 to $4.5 billion per annum while in Morocco the estimated national costs (direct and indirect) are in the range $5.9 to $8.2 billion per annum” [28]. Two types of costs were cited as barriers for lifestyle intervention change. The first was the direct cost such as medical and nonmedical costs that included pharmaceutical costs, followed by diagnostic and investigation costs, medical equipment supply costs, and consultation costs. The second type of cost includes indirect costs were those costs associated with the loss of income, disability, and premature mortality [28].

#### 4.1.3. Poverty

Poverty (economic constraint) is another barrier for type 2 diabetes prevention and management discussed in the reviewed literature. Most Africans live under the poverty line and get food for the survival of their life. They eat whatever is available and affordable. The most widely used crops in the region include rice, teff, maize, and wheat that are high carb diets served on each meal (Rawal et al., 2018). In addition, most urban populations consume the less healthy options like prepacked foods.

Poverty includes lack of access to health care and to follow the diabetic diet recommended by health care professionals was a challenge for those in the region [33]. Economic constraints present challenges for many of the health systems in Africa due to drug costs, diagnostic costs, and consultation costs that are an additional burden for diabetes patients [28]. Studies in Senegal, Uganda, and Zimbabwe showed that financial constraint is one of the main barriers to proper glycemic control in most African countries [29].

Limited health infrastructures in developing countries like Africa meant poor access to health services for diabetes patients. These limited infrastructures and human resources included pharmaceutical supplies, diabetes specialists or endocrinologists, shortage of laboratory supplies, and screening reagents including anhydrous glucose [30].

#### 4.1.4. Population Changes

Population changes are driving forces in the rapid increase of the number of individuals with diabetes in Africa. Most Africans who previously lived in rural areas migrated into urban ones and adopted western lifestyle habits such as unhealthy dietary patterns and inactivity [28]. The lifestyle and dietary habits in low-income countries are changing towards risky behavior such as physical inactivity due to increased use of motorized transportation, and urbanization that changed people's eating habits toward fast-food [25].

### 4.2. Strategies to Overcome Barriers to Lifestyle and Dietary Pattern Changes and Intervention in Africa

According to WHO [44], the aim of strategies for preventing and managing type 2 diabetes include “to promote primary, secondary, & tertiary prevention interventions in favor of diabetes; to improve the capacities of heath personnel to better deal with diabetes and associated diseases; to support research in community interventions, including traditional medicine” (p.4). Strategies to overcome the barriers of lack of knowledge and education, poverty and cost, population changes, and lack of access to healthcare are discussed below.

#### 4.2.1. Health Education

The most commonly cited strategies to overcome the barriers to lifestyle and dietary pattern changes and interventions are providing diabetes health education programs through coordinating the community and health extension providers, mobilizing community resources, and providing diabetes-related health care education in the community. For example, solid coordination and collaboration is essential, as patients have to circulate between different services and might even have to consult various professionals [34]. The other commonly cited strategy to overcome the education barrier was creating community awareness regarding chronic illnesses such as HTN and DM and encouraging screening activities through the collaboration of health care providers, community workers, and village health care team members [35]. Health education programs are also important for correcting patients' misperceptions about the diseases.

Health education must be culturally relevant. Through intensified diabetes self-management education and culturally sensitive diabetes education by community healthcare professionals and village healthcare teams the barriers to self-care and complications and death from diabetes will be reduced. “Collaboration with the civil society being involved in the fight against diabetes, community participation, gender sensitivity and consideration of local beliefs as necessary to generate awareness on diabetes is important to overcome the barrier to prevent and manage diabetes” [44].

#### 4.2.2. Cost

Diabetes imposes a considerable economic burden on individuals. The largest burden on diabetic patients was the cost of drugs [28]. “The healthcare policies should focus on a reduction in the burden of diabetes on individuals and the promotion of prescriptions of diabetes drugs in their generic names. The adoption of policies targeted at a reduction in harmful alcohol use, tobacco consumption and physical inactivity will contribute to a reduction in Non Communicable Disease (NCD) prevalence and therefore healthcare costs” [28]. The drug cost can be minimized through encouraging government and private sectors to build local pharmaceutical industries to minimize the high cost of importing drugs.

#### 4.2.3. Access to Healthcare

According to Kenya National Diabetes Strategy (KNDS), advocacy is one of the key strategies in addressing the lack of access to healthcare. “Making diabetes everybody's business combines the notion of individual, community, social, corporate and government responsibility. This is because diabetes affects everybody in some way and so should be the responsibility of everyone to address the determinants of diabetes and related chronic diseases and conditions” [45]. Health service availability plays an important role in lifestyle modifications. Governmental bodies and stakeholders should integrate to improve healthcare service availability and accessibility. The second important strategy is capacity building—“this is about making what you have, work better by improving the human resource capacity, making health systems more responsive to diabetes through improved infrastructure, adequate medical supplies, improved diagnostic services, and provision of necessary protocols and standards” [45].

#### 4.2.4. Population Changes

Acknowledging population change and providing access to health care in both rural and urban areas of Africa will help to bridge the gap. Culturally adaptable health education intervention programs for most African regions would be the appropriate strategy to overcome the barriers due to epidemiological transition in order to prevent and manage diabetes [46]. This culturally sensitive education must reach rural areas as well as urban ones and can help the society to maintain and adopt healthy lifestyle and dietary habits.

## 5. Conclusion

Lifestyle interventions including regular physical exercise, weight management, and adhering to health care professionals' recommendations on a healthy diet are the cornerstone of the prevention and management of diabetes. Healthy diets such as the Mediterranean diet and eating lower carbohydrates and fat and increased fruits and vegetables are important for diabetes prevention and management. Creating diabetes support groups and continuing public education on diabetes through religious institutions increases the awareness of diabetes in the society. Therefore, focusing on lifestyle modification and dietary pattern is the key to prevent and manage type 2 diabetes.

Lack of knowledge, poverty (economic constraints), cost of diabetes care, and lack of access to healthcare are the main barriers to adherence to prevent and manage diabetes in Africa. The strategies for the barriers include health education programs, advocacy, and capacity building and are key to overcoming the barriers to adherence to lifestyle modification that assist in the prevention and management of type 2 diabetes.

Health professionals working in Africa should be at the forefront to provide health education to promote the health of diabetes patients by addressing the systemic barriers such as (population changes, poor access, western cultural influences, and low-quality healthcare) and personal barriers such as (poverty, cost, educational status, and perceptions about the disease). Solid coordination and collaboration between healthcare professionals, government, nonprofit organizations, and the society has a significant impact to create awareness for the prevention and management of diabetes in Africa.

## Figures and Tables

**(a):** 

Citation/level and quality	Purpose	Sample/setting	Design	Results/conclusion	Recommendations
Ganiyu et al., [29] Johns Hopkins level: III Quality: high quality	To investigate the barriers for nonadherence to the recommended diet and exercise among type 2 diabetes mellitus patients	The sample size was 96 with age greater than 30 years over a period of 3 successive months. Extension II clinic, in Gaborone, Botswana.	Institutional-based cross-sectional study. Data was collected using self-administered questionnaire	The main barriers for nonadherence to the recommended diet were poor self-discipline, lack of awareness, eating outside home, economic constraints and lack of self-control. The main barriers for nonadherence to the recommended exercise were poor knowledge, perceived exercise as potentially exacerbating illness, lack of exercise partner, specific locations away from home, and winter weather.	Awareness creation regarding the effectiveness of diet and exercise should be delivered through diabetes education program using different motivational interviewing models. Multiple strategies such as creating support group and increasing awareness programs for diet and exercise recommendations should be included in primary health care.

**(b):** 

Citation/level and Quality	Purpose	Sample/setting	Design	Results/conclusion	Recommendations
Ayele et al., [30] Johns Hopkins level: III Quality: high quality	To examine the barriers for dietary adherence among patients with type 2 diabetes	Patients with T2DM aged >18 years who visited the hospital for follow-up from August 1–October 30–2017 In Debre Tabor General Hospital, Northwest Ethiopia	Institutional-based cross-sectional study. The Perceived Dietary Adherence Questionnaire (PDAQ) tool was used for dietary adherence measurement	Nonadherence to recommended diet among T2DM is high. 74.3% of the patients reporting nonadherence. The barriers for the high rate of poor adherence were poor knowledge, lack of education on diet, and financial constraints on the recommended diet, low income, lack of previous exposure to dietary education and the presence of other chronic illness.	Health workers should become effective in addressing these barriers through guiding and teaching patients. Health care decision makers should follow effective dietary guidelines for people with T2DM.

**(c):** 

Citation/level and quality	Purpose	Sample/setting	Design	Results/conclusion	Recommendations
Assaad-Khalil et al., [31] Johns Hopkins level: III Quality: high quality	To investigate the potential barriers to diabetes care delivery in the Middle East and South Africa.	One thousand and eighty-two physicians Countries in the Middle East and Africa (Egypt, Kingdom of Saudi Arabia, United Arab Emirates, South Africa, and Lebanon)	Descriptive cross-sectional study.	Poor lifestyle adherence, illiteracy, and patients' poor diet were the main barriers for diabetes prevention and management.	Future research should be conducted to numerically measure the impact of these barriers on the delivery of diabetes care.

**(d):** 

Citation/level and quality	Purpose	Sample/setting	Design	Results/conclusion	Recommendations
Muhabuura, [32] Johns Hopkins level: III Quality: high quality	To determine the knowledge of the role of diet and exercise lifestyle recommendations, prevalence of nonadherence to diet and exercise recommendations, and describe the factors associated with nonadherence to diet and exercise among type 2 diabetic patients	The total sample size was 324 participants with age greater than 30 years In Kenyatta National Hospital (KNH) diabetic clinic	Descriptive cross- sectional study A structured, pilot-tested questionnaire was used to collect data.	The results from this study demonstrated lower rates of nonadherence to diet and exercise recommendations among people diagnosed with type 2 diabetes mellitus Financial constraints and lack of detailed written instructions regarding diet as reasons for nonadherence to diet while reasons for nonadherence to exercise were lack of information and belief that exercise potentially exacerbates illness.	There is a need for active involvement of family and friends of diabetic patients in management of type 2 diabetes. Detailed written instructions on proper diet and exercise should be tailored to individual patients taking into account other factors such as presence of other co morbidities.

**(e):** 

Citation/level and quality	Purpose	Sample/setting	Design	Results/conclusion	Recommendations
Schellenberget al., [23] Johns Hopkins level: I Quality: high quality	To review the importance of lifestyle interventions on decreasing progression to diabetes in high-risk patients or progression to complications such as (cardiovascular disease and death).	Nine RCTs on at risk for diabetes and eleven on patients who had diabetes Publications conducted in patients with type 2 diabetes in the world	Systematic review and meta-analysis of RCTs.	Comprehensive lifestyle interventions including diet and physical exercise effectively decrease the incidence of type 2 diabetes. There was no evidence on reducing mortality in patients who already have type 2 diabetes and insufficient evidence on reducing complication	The review included only RCTs studies. A review of cohort studies is advantageous to provide data on the effect of different lifestyle interventions

**(f):** 

Citation/level and Quality	Purpose	Sample/setting	Design	Results/conclusion	Recommendations
Rawal, et al. [42] Johns Hopkins level: I Quality: high quality	To review the cost-effectiveness of nonpharmacological interventions aimed at preventing T2DM and its related complications in developing countries such as Africa and Asia.	Nine studies were included in this review Publications conducted in patients with type 2 diabetes in developing countries.	Systematic review of RCTs.	The result of the review showed a significant reduction in the development of T2DM in the intervention group compared with controls. The study comprised three lifestyle intervention groups (diet, exercise, and diet + exercise), with one control group with no life style intervention measures.	Future programs should focus on the appropriate development, adaptation, and implementation of efficacious, cost-effective intervention methods through health education and promotion, and creating support group.

**(g):** 

Citation/level and quality	Purpose	Sample/setting	Design	Results/conclusion	Recommendations
Ayele, et al. [26] Johns Hopkins level: III Quality: good	To identify predictors of self-care behaviors among patients with diabetes.	Two hundred twenty two T2DM patients. Three hospitals in Harar town, Ethiopia.	Quantitative cross-sectional study	Patients with poor information were less likely to adhere to diabetes self-care. Patients, who were more educated, middle income, had high-perceived severity of diabetes and less perceived barrier to self-care were more likely to take diabetes self-care.	To increase the awareness on self-care behavior, diabetes education should focus on severity of diabetes and how to overcome the barriers for self-care.

**(h):** 

Citation/level and quality	Purpose	Sample/setting	Design	Results/conclusion	Recommendations
Alouki, et al. [24] Johns Hopkins level: I Quality: high quality	To investigate key findings of economic evaluations of lifestyle interventions for the primary prevention of type 2 diabetes (T2D) in high-risk subjects	20 articles were included. 7 were RCTs and 13 using modeling techniques. Publications conducted in patients with type 2 diabetes since January 2009.	Systematic review of RCTs	The importance of lifestyle interventions combining diet and physical activity to prevent diabetes in at-risk population groups.	Lifestyle interventions should be further stressed as an effective strategy to prevent or delay diabetes through creating effective and efficient diabetes education.

**(i):** 

Citation/level and quality	Purpose	Sample/setting	Design	Results/conclusion	Recommendations
Tewahido & Berhane [27] Johns Hopkins level III Quality: high quality	To describe self-care practices among individuals with type II diabetes in Addis Ababa, Ethiopia.	Type 2 diabetes between the ages of 35-65 years that came to follow up clinics from November 2013 to February 2014. A purposive sampling procedure Type II diabetes patients in Addis Ababa, Ethiopia.	A qualitative method of descriptive cross-sectional study	The review showed that self-care habits of patients were not adequate. Most of the patients were inadequately adhered to the recommended dietary and physical exercise. Diabetes patients mainly dependent on prescribed medications to control their glycemic level.	Attention should be given to improve patient diabetes self-management education and support to reduce diabetes related complications.

**(j):** 

Citation/level and quality	Purpose	Sample/setting	Design	Results/conclusion	Recommendations
Rahati, et al. [25] Johns Hopkins level II Quality: high quality	To investigate evidence regarding epidemiologic and clinical trial.	Systematic review of articles. Publications conducted in patients with type 2 diabetes in Middle east and Africa	Systematic review without meta-analysis	The lifestyle and dietary habits in low-income countries are changing towards risky behavior such as physical inactivity due to use of transportation, and urbanization that change people eating habit towards fast-food.	Interventions should be specified for each age group category and their developmental stages.

**(k):** 

Citation/level and quality	Purpose	Sample/setting	Design	Results/conclusion	Recommendations
Mutyambizi, et al. [28] Johns Hopkins level II Quality: high quality	To investigate the cost of diabetes in Africa.	Systematic review of twenty six articles were reviewed Publications conducted in Africa	Systematic review	The diabetes related direct costs differed between countries. The most commonly listed healthcare costs were pharmaceutical costs, followed by diagnostic and investigation costs, medical equipment supply costs and consultation costs. Estimation of the costs associated with diabetes is important to achieve the targets in SDG3 set by 2030.	Future research should focus on increasing the transparency and methodological principles of cost of illness studies.

**(l):** 

Citation/level and quality	Purpose	Sample/setting	Design	Results/conclusion	Recommendations
Belue, et al. [33] Johns Hopkins level: III Quality: good quality	To examine diabetes patients experience on diabetes self-management among clinic patients.	195,000 patients were included in the Grand M'bour Medical Clinic during the summer of 2009 and 2010. The Grand M'bour Medical Clinic in Senegal.	Qualitative study with the PEN-3 model using semistructured interview. The PEN-3 model consists of three interrelated domains: relationships, expectations, cultural empowerment, and cultural identity.	Lack of adequate finance to access health care and to follow the recommended diabetic diet by the health care professionals were the main barriers to diabetes management. Family and significant others have significant positive and negative role in the management of diabetes.	Since participants were only those who seek health care, those who do not have access to health care were not included. Further approach should be investigated to include all patients and other studies should be conducted using objective measures of diabetes control to avoid bias.

**(m):** 

Citation/level and quality	Purpose	Sample/setting	Design	Results/conclusion	Recommendations
Mendenhall and Norris [34] Johns Hopkins level: III Quality: good quality	To examine opportunities and challenges of diabetes care of low-income women in South Africa.	27 women diabetic patients were included In Chris Hani Baragwaneth Hospital in Soweto, South Africa.	Qualitative study conducted through 27 face to face interview of patients	Lack of education on diabetes, not following diabetes treatment routines, structural barriers to clinics, and poor access to medications are the main barriers of diabetes management with patients. Lack of continuous diabetes counseling, drug availability, quality of care, and patient wait times affect patient frustration and lack of confidence in the public health care system	Community-based care by mobilizing community resources and further health care education is necessary for better control of diabetes.

**(n):** 

Citation/level and quality	Purpose	Sample/setting	Design	Results/conclusion	Recommendations
Chang, et al. [35] Johns Hopkins level: III Quality: good quality	To investigate the challenges to hypertension and diabetes care in rural Uganda.	16,694 adults' patients were included from different clinical departments. Conducted in Nakaseke District, Uganda.	Qualitative study with semi-structured face-to-face in-depth interviews were conducted	The main barriers for nonadherence to diabetes treatment are poor knowledge regarding the prevention of chronic diseases such as HTN and DM, patients' mistrust of the healthcare system, and skepticism from health care professionals (HCPs) and village health-care team (VHTs').	Further education should be delivered concerning HTN and DM Screening activities through the collaboration of health care providers, community workers, and village health care team is useful to overcome the barrier.
